# Antiproliferative and cytotoxic activity of Geraniaceae plant extracts against five tumor cell lines

**DOI:** 10.2144/fsoa-2021-0109

**Published:** 2021-12-03

**Authors:** Shynggys Sergazy, Anastassiya Vetrova, Ilkay Erdogan Orhan, Fatma Sezer Senol Deniz, Ahmet Kahraman, Jian-Ye Zhang, Mohamad Aljofan

**Affiliations:** 1 National Laboratory Astana, Nazarbayev University, Nur-Sultan, 010000, Kazakhstan; 2 Department of Biomedical Science, Nazarbayev University School of Medicine, Nur-Sultan, 010000, Kazakhstan; 3 Department of Pharmacognosy, Gazi University, Ankara, 06330, Turkey; 4 Department of Biology, Usak University, 64200 Usak, Turkey; 5 Key Laboratory of Molecular Target & Clinical Pharmacology & the State Key Laboratory of Respiratory Disease, School of Pharmaceutical Sciences & The Fifth Affiliated Hospital, Guangzhou Medical University, Guangzhou, P.R. China

**Keywords:** anticancer, antiproliferative, breast cancer, cytotoxicity, *Erodium*, Geraniaceae, *Geranium*

## Abstract

**Aim:**

To determine the antiproliferative and cytotoxic activities of *Geranium* and *Erodium* species against human cancer and noncancer cell lines, respectively.

**Methods:**

Twenty-one species of *Geranium* and *Erodium* were extracted and screened against cancerous and noncancerous human cell lines.

**Results:**

In a dose-response manner, *G. glaberrimum*, *G. asphodeloides*, *E. brandianum* and *E. leucanthum* were able, with variable potency, to inhibit cellular proliferation. Except for *E. brandianum*, all extracts induced cellular autophagy in tumor cells with similar levels to that of rapamycin; but, only *E. brandianum* induced cellular apoptosis, likely through Bcl2 and BAX protein expressions.

**Discussion:**

This is the first study to report the potential antiproliferative effects of ethanol extracts of several Geraniaceae species.

## Lay abstract

Several plant species of the genera *Geranium* and *Erodium* were extracted and screened against five tumor cell lines for their potential antitumor activities. Out of the tested species, four showed potential antitumor action against all the tumor cells used. Interestingly, they appeared to achieve their antitumor activities by inducing important cellular defense mechanisms called autophagy and apoptosis. The current work is the first to test the antiproliferative potential of these species against different tumor cells.

Anticancer drugs are considered the first line of treatment for some types of cancer [1]. There are several anticancer drugs with different origins and mechanisms of action available to treat certain types of cancers, but the majority of these drugs cause serious side effects. For instance, alkylating agents, topoisomerase inhibitors and antimicrotubule agents directly target DNA replication and cell division in cancer and normal cells, leading to serious side effects [2–4]. Therefore, there is a need to develop new anticancer drugs with fewer side effects. Medicinal plants have always been a valuable source of therapeutic agents and drug leads, including anticancer drugs. It was estimated that 60% of all chemotherapeutic agents approved by the FDA, including vincristine, vinblastine and taxol, trace their origin to natural products or their derivatives [5]. Natural-based drugs are those that contain pharmacologically active ingredients derived from biological or mineral sources and intended for use in the diagnosis, cure, mitigation and prevention of disease [6].

*Geranium* L. (Geraniaceae) is a large genus comprising almost 400 plant species [7] and was traditionally used for the treatment of various illnesses, including diabetes, hemostasis, diarrhea, cough, whooping cough, kidney pain, tonsillitis and gastrointestinal ailments [8]. However, some studies suggest that the *Geranium* species is potentially cytotoxic [9–12]. Another major plant genus belonging to Geraniaceae is *Erodium* L'Herit which consists of 74 species predominantly found in the Mediterranean region [13]. Many of the member species were reportedly used in folk medicines in various countries to treat infections, wounds, hepatitis, constipation, coagulation, hemorrhoid, toothache, flu and diabetes [14]. In addition, some *Erodium* species were shown to have cytotoxic activity against MT-4, MCF7 and Madin–Darby bovine kidney cells [15,16]. The two genera are also known to have rich phytochemical compositions, particularly polyphenols [12]. The latter was shown to affect cellular apoptosis and senescence processes, which are considered vital processes for cancer proliferation; however, their effectiveness appeared to be cell-type dependent [17].

The ethnopharmacological, bioactivity and phytochemical data on these species demonstrate that the two genera, namely *Geranium* and *Erodium*, represent a potentially rich source of biologically active components. While some of their biological activities have been reported, their antiproliferative potential has not been explored. Thus, it is hypothesized that screening of *Geranium* and *Erodium* species against tumor cells might shed new light on the therapeutic potential of these genera. The current study aims to evaluate the potential *in vitro* antiproliferative activity and cytotoxicity of ethanol extracts from *Geranium* and *Erodium* genera using cell-based assays against several cancer and noncancerous cell lines, respectively. *In vitro* cell-based assays have been extensively used as a tool for *in vitro* drug screening, including anticancer screening, as they enable the rapid determination of potential antiproliferative activity. Therefore, screening plant extracts using a reliable drug screening methodology may result in the identification of effective, less toxic and novel anticancer drug leads.

## Methods

### Plant materials & extraction procedure

Plant materials were extracted using the maceration method with ethanol (95%), which is a general extraction technique for medicinal plants. Briefly, powdered plant materials were placed with a solvent in a stoppered container at room temperature for at least 3 days. When the dissolution of the soluble phytochemicals was complete, the liquid part was separated by filtration [18]. In total, 21 ethanol extracts were prepared from *Erodium absinthoides*: Willd. subsp. *absinthoides* (four locations), *E. amanum* Boiss. and Kotschy, *E. birandianum* Ilarslan and Yurdakulol, *E. cedrorum* Schott and Kotschy ssp. *cedrorum*, *E. ciconium* (L.) L'Herit., *E. cicutarium* (L.) L'Herit., *E. gruinum* (L.) L'Herit., *E. hendrikii* Alpinar, *E. leucanthum* Boiss., *E. malacoides* (L.) L'Herit., *E. pelargoniiflorum* Boiss. and Heldr., *E. somanum* H. Peşmen as well as *Geranium asphodeloides* Burm., *G. lasiopus* Boiss. and Heldr., *Geranium glaberrimum* Boiss. and Heldr (two locations), *G. lucidum* L., *G. purpureum* Vill. and *G. tuberosum* L. (two locations). Locations and dates of collection as well as herbarium numbers for all plant species screened are shown in Table 1. The plant samples were collected, identified and preserved in a private herbarium collection at the Scientific Analysis Technological Application and Research Center (Usak University, Turkey). The aerial parts of the collected plant samples were cut into small pieces and dried at room temperature. The dried pieces were ground to a fine powder using a mechanical grinder. The dried and powdered aerial parts of the plants were extracted with 95% ethanol (EtOH) over a 5-day period with casual manual stirring. The EtOH phases of each macerate were independently filtered using Whatman filter paper (150 mm) and concentrated under vacuum in a rotary evaporator (Büchi, Switzerland) to yield the crude extracts. The extracts were preserved in a deep freezer until the experiments.

**Table 1.** Collection locations, dates and herbarium numbers for the screened plants.

Species	Endemism (E)	Collection location, date and herbarium number
*Erodium absinthoides* Willd. subsp. absinthoides (Trautv.) Davis (no. 1)	E	Ankara province, Ayaş Mountain, June 2014, A Kahraman 1824
*Erodium absinthoides* Willd. subsp. *armenum* (Trautv.) Davis (no. 2)	–	Erzincan province, North of Sipikör Mountain, July 2014, 2472 m, A Kahraman 1872
*Erodium absinthoides* Willd. subsp. *armenum* (Trautv.) Davis (no. 3)	–	Erzincan province, Sipikör pass on Sipikör Mountain, July 2014, A Kahraman 1873 (a)
*Erodium absinthoides* Willd. subsp. *haradjianii* (Davis) Davis (no. 4)	E	Osmaniye province, Düziçi, Düldül Mountain, September 2014, T Dirmenci
*Erodium amanum* Boiss. and Kotschy	E	Hatay province, Belen, Çobandere, June 2014, A. Kahraman 1854
*Erodium birandianum* Ilarslan and Yurdakulol	E	Kastamonu province, Devrekani, Yaraligöz Mountain, July 2014, A Kahraman 1859, 1830 m
*Erodium cedrorum* Schott and Kotschy subsp. *cedrorum*	E	Adana province, Aladag, Kicak village, Tahtali upland, June 2014, A Kahraman 1855
*Erodium ciconium* (L.) L'Herit.	–	Isparta province, Isparta to Antalya, 10 km to Elsazi, May 2014, A Kahraman 1768
*Erodium cicutarium* (L.) L'Herit.	–	Kütahya province, 15 km from Gediz to Kütahya, May 2014, A Kahraman 1810
*Erodium gruinum* (L.) L'Herit.	–	Antalya province, Akseki to Manavgat, Murtiçi, May 2014, A Kahraman 1786
*Erodium hendrikii* Alpinar	E	Gümüşhane province, Yagmurdere, July 2014, A. Kahraman 1893
*Erodium leucanthum* Boiss.	E	Denizli province, Babadag, above Saçmagedigi, May 2014, A Kahraman 1815
*Erodium malacoides* (L.) L'Herit.	–	Antalya province, Akseki to Manavgat, Murtiçi, May 2014, A Kahraman 1783
*Erodium pelargoniiflorum* Boiss. & Heldr.	E	Isparta province, Sütçüler, Kuzca village, 1 km to Tota, June 2014, A Kahraman 1825
*Erodium somanum* H. Peşmen	E	Manisa province, Soma, above Kızılören village, Sifa Mountain, May 2014, A Kahraman 1798
*Geranium asphodeloides* Burm.	–	Kastamonu province, Ilgaz Mountain National Park, July, 2014, A Kahraman 1858
*Geranium lasiopus* Boiss. and Heldr.	E	Antalya province, Akseki Güzelsu, June 2016, A Kahraman 2397
*Geranium glaberrimum* Boiss. and Heldr. (no. 1)		Konya province, Ibradi-Derebucak road, near Derebucak, 23.04.2016, A Kahraman 2308
*Geranium glaberrimum* Boiss. and Heldr. (no. 2)	E	Antalya province, Akseki, Sadiklar village, May 2014, A Kahraman 1781
*Geranium lucidum* L.	–	Konya province, Ibradi-Derebucak road, near Derebucak, 23.04.2016, A Kahraman 2309
*Geranium purpureum* Vill.	–	Antalya province, Köprülü canyon, 22.04.2016, A Kahraman 2298
*Geranium tuberosum* L. (no. 1)	–	Antalya province, Elmalı, near Uzungeriş hill, June 2014, A Kahraman 1838
*Geranium tuberosum* L. (no. 2)	–	Antalya province, Akseki to Beyşehir, 54 km to Beyşehir, May 2014, A Kahraman 1777

subsp.: Subspecies.

### *In vitro* screening

#### Cell lines

Breast cancer cells MCF-7 (a gift from the Tulchinsky lab), noncancerous fibroblastic cells NIH/3T3 (a gift from Carmen Birchmeier's lab), muscle Ewing's sarcoma cells A-673 (ATCC^®^ CRL-1598™, USA), epithelial cervical carcinoma DoTc2 4510 (ATCC^®^ CRL-7920™, USA), hepatocellular carcinoma HUH-7 (gifted by the National Laboratory, Astana) and malignant melanoma A-375 (ATCC^®^ CRL-1619™, USA) cell lines were cultured in normal glucose Dulbecco's modified Eagle's medium (DMEM, Sigma, MO, USA) supplemented with fetal bovine serum (FBS; 10% [v/v]; Sigma) and streptomycin and gentamycin (1% [v/v]; Sigma). Cells were incubated in a humidified atmosphere at 37°C and 5% CO_2_.

### Antiproliferative & cytotoxicity assays

#### Cellular proliferation assay

Cellular proliferation was determined using the 3-[4,5-dimethylthiazol-2-yl]-2,5-diphenyltetrazolium bromide (MTT) method [19]. Briefly, cells were seeded at 1 × 10^4^ cells/mL in 96-well microtiter plates in minimum essential medium with FBS and incubated overnight for attachment. A final volume of 100 μl of serial log dilutions of different test compounds (blind screening) was added to each well in triplicate (final compound concentrations ranged from 20 ng/ml to 200 μg/ml). Cells were incubated in the presence of different investigational drugs at 5% CO_2_ at 37°C for 24 h. Cells were then treated with MTT (Sigma, MO, USA) and incubated for 4 h. After the incubation period, formazan crystals were dissolved by MTT solubilization solution (Sigma) and the absorbance was measured at 570 nm using a 96-well imaging reader (Cytation™ 5, Bio-Tek Instruments, Inc., USA). The cytotoxicity index was determined using the untreated cells as the negative control. The percentage of antiproliferative activity (in cancer cells) and cytotoxicity (in noncancerous cells) were calculated according to the method described by Florento *et al.* using the treated reading/negative control (nontreated); cell viability (%) = [(A_sample at 570 nm_/A_control at 570 nm_)] × 100 [20].

#### Therapeutic index

The therapeutic index (TI) for each of the tested extracts was determined by dividing the extract's IC_50_ by its CC_50_, which was determined using dose-response and dose-cytotoxicity graphs, respectively. Briefly, the extract concentration that inhibited cancer cell proliferation by 50% (IC_50_) was divided by the extract concentration that reduced the cellular viability of noncancerous cells by 50% (CC_50_). Both values were determined by plotting data points for a range of drug concentrations and calculating the values using regression analysis.

### Autophagy assay

Cellular autophagy was measured using an Autophagy Assay kit (Abcam, UK) according to the manufacturer's guidelines. Cells were cultured in a 96-well black plate with a clear bottom at a density of 1 × 10^4^ cells/ml, then treated with the extracts at different concentrations (final concentrations 20 ng/ml to 200 μg/ml), and incubated at 5% CO_2_ at 37°C for 24 h. Following the incubation period, the medium was removed from the cells and 100 μl of the autophagosome detection reagent working solution were added to each well (samples and controls), and then incubated at 37°C with 5% CO_2_ for 30 min. Cells were washed three times by gently adding 100 μl of wash buffer to each well and was then carefully removed to prevent dislodging the cells. Fluorescence intensity was then measured using an imaging reader (Cytation^TM^5, Bio-Tek Instruments, Inc., USA) at λ_ex360_/λ_em520_ nm.

### Apoptotic protein expression using western blot

MCF-7 cells were incubated for 24 h and then treated with doxorubicin (1 uM) alone or cotreated with 200 µg/ml of *E. birandianum*. After 48 h of treatment, the cells were harvested and lysed using loading dye containing 1% Triton X-100, 50 mM Tris-Cl (pH: 6.8), 100 mM NaCl 1% SDS, 10% glycerol, β-mercaptoethanol and bromophenol blue. Protein concentrations were determined and equal quantities of protein (40–50 μg) were loaded on 12% SDS-PAGE and transferred to polyvinylidene fluoride (PVDF) membranes. The membranes were incubated in a blocking buffer (Tris-buffered saline [TBS] buffer containing 0.1% Tween 20 [Sigma] and 5% nonfat dry milk) for 1 h at room temperature to block nonspecific binding. The membranes were then incubated with primary antibodies, anti-β actin, anti-BAX and anti-BCL2 at 4°C overnight. The membranes were washed in TBS, and incubated with horseradish peroxidase (HRP)-conjugated secondary antibodies for 1 h at room temperature and then detected using an enhanced chemiluminescence (ECL) detection system. For band intensity comparisons and quantification, NIH ImageJ software was used (http://rsb.info.nih.gov/ij/).

### Statistical analysis

All data are expressed as mean ± standard error of the mean unless otherwise stated. Data were analyzed using one-way analysis of variance (ANOVA) and Student's *t*-tests. Results with p-values less than 0.05 were considered statistically significant.

## Results

### Antiproliferative & cytotoxic activity

**Figure 1.**
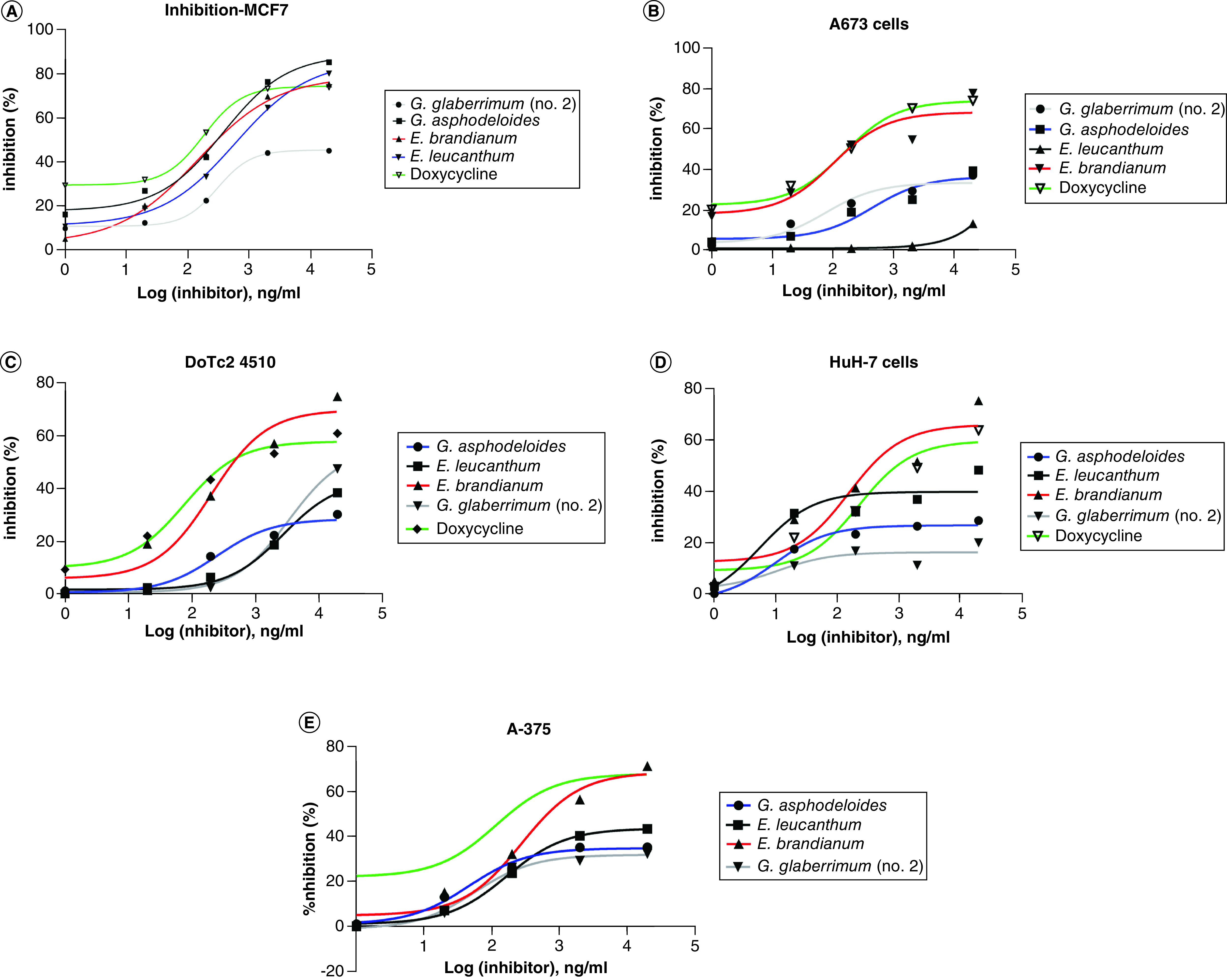
Inhibition of cellular proliferation. Inhibition of cellular proliferation following 24 hours treatment with serial concentrations of doxycycline, *Geranium glaberrimum*, *Erodium brandianum*, *Erodium leucanthum* and *Geranium asphodeloides*. The inhibition of cellular proliferation was measured in **(A)** MCF-7, **(B)** A-673, **(C)** Do-TC2 4510, **(D)** HUH-7 and **(E)** A-375 cells. The results shown are the average of three independent experiments.

**Figure 2.**
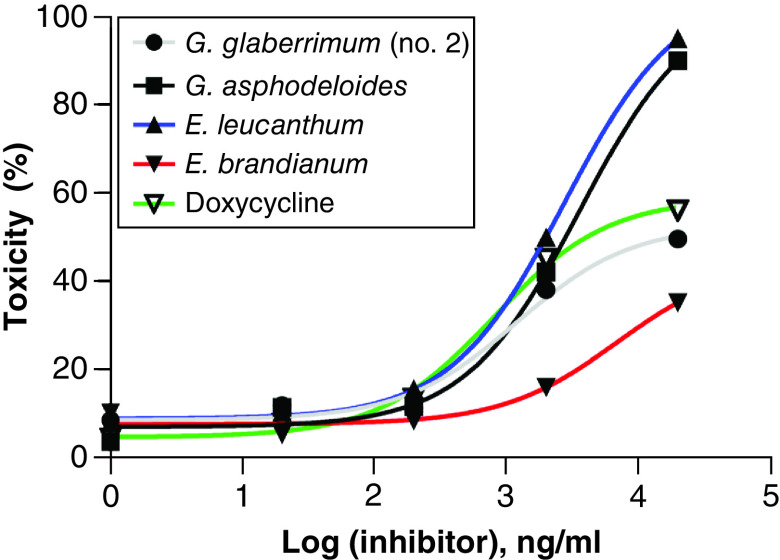
Cellular toxicity. The potential cellular toxicity of four active plant extracts and doxycycline were measured using noncancerous fibroblastic cell line, NIH/3T3. Cells were treated with serial concentrations of each of the extracts for 24 hours. The results shown are the average of three independent experiments.

A total of 21 plant extracts were evaluated in a blind manner for their potential antiproliferative activity against five cell lines: human breast cancer cell lines (MCF7), A673 muscle Ewing's sarcoma, 4510 epithelial cervical carcinoma, HuH7 hepatocellular carcinoma and A375 malignant melanoma. The results showed that 10 extracts displayed antiproliferative activity at high concentrations (200 μg/ml). Interestingly, four of the tested extracts belonging to *G. asphodeloides*, *G. glaberrimum* (no. 2), *E. leucanthum* and *E. birandianum* inhibited cellular proliferation in a dose-response manner with different potency against different cancer cell lines (Figure 1A–E). However, in order to determine the cytotoxicity of the extracts and to confirm their antiproliferative specificity, the extracts were screened again using noncancerous NIH/3T3 cells. Of the extracts screened, *E. birandianum* was shown to be significantly less toxic than the others (Figure 2).

### Therapeutic index

The IC_50_ and CC_50_ values for each of the tested extracts were determined by the dose-response curves shown in Figures 1A–E & 2, respectively. As shown in Table 2, *E. birandianum* had the highest TI in all tested cell lines, which was much higher than doxycycline (positive control; Table 2). The TI for *E. birandianum* extract was found to be significantly higher than the other three extracts in all of the treated cells, but significantly greater than the positive control in HUH7 (α <0.05) and A375 (α = 0.04) cells only.

**Table 2.** Therapeutic indices for the tested extracts.

Cell line	*G. glaberrimum* (no. 2)	*G. asphodelioides*	*E. leucanthum*	*E. birandianum*	Doxycycline
MCF-7	1.9	2.2	2.8	6.0	4
A673	1.2	1.3	1.0	6.5	3.5
4510	1.8	1.0	1.5	5.5	3.8
HUH7	–	0.9	1.6	5.9	2.7
A375	1.9	0.9	1.5	5.2	3.3

### Autophagy assay

**Figure 3.**
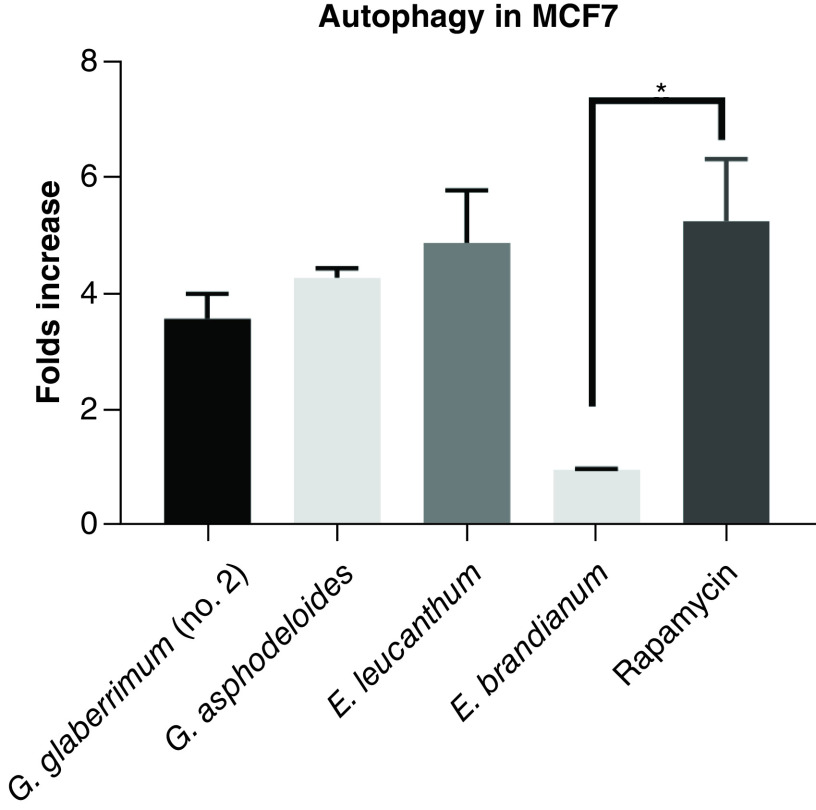
Induction of autophagy. The autophagy induction abilities of the four tested active plant extracts were compared with the positive control, rapamycin. MCF-7 cells were treated with each of the plant extracts for 24 h. The results show a fold increase over the relevant negative control (no treatment). *Statistically significant difference (p < 0.05). The results shown are the average of three independent experiments.

To determine the potential antiproliferative mechanisms of the extracts, the concentration of IC_50_ for each extract was selected and screened in MCF7 cells, since it is the most sensitive cell line for these extracts as determined by the TI values. The active extracts, except for *E. birandianum*, induced autophagy in MCF7 cells with variable potency, but not in the noncancerous fibroblastic cell lines (NIH/3T3; Figure 3). At a final concentration of 2 μg/ml, the extract from *E. leucanthum* induced a 5-fold increase in autophagy compared with nontreated cells; the *G. asphodeloides* extract induced an approximately 4.2-fold increase compared with nontreated cells; and *G. glaberrimum* (no. 2) induced autophagy at approximately 3.8-times that of the nontreated cells. However, no significant difference was observed between the autophagy inducer (rapamycin) and the extracts of *G. glaberrimum* (no. 2; α = 0.06), *G. asphodeloides* (α = 0.18) and *E. leucanthum* (α = 0.65; Figure 3).

### Apoptosis protein expression

**Figure 4.**
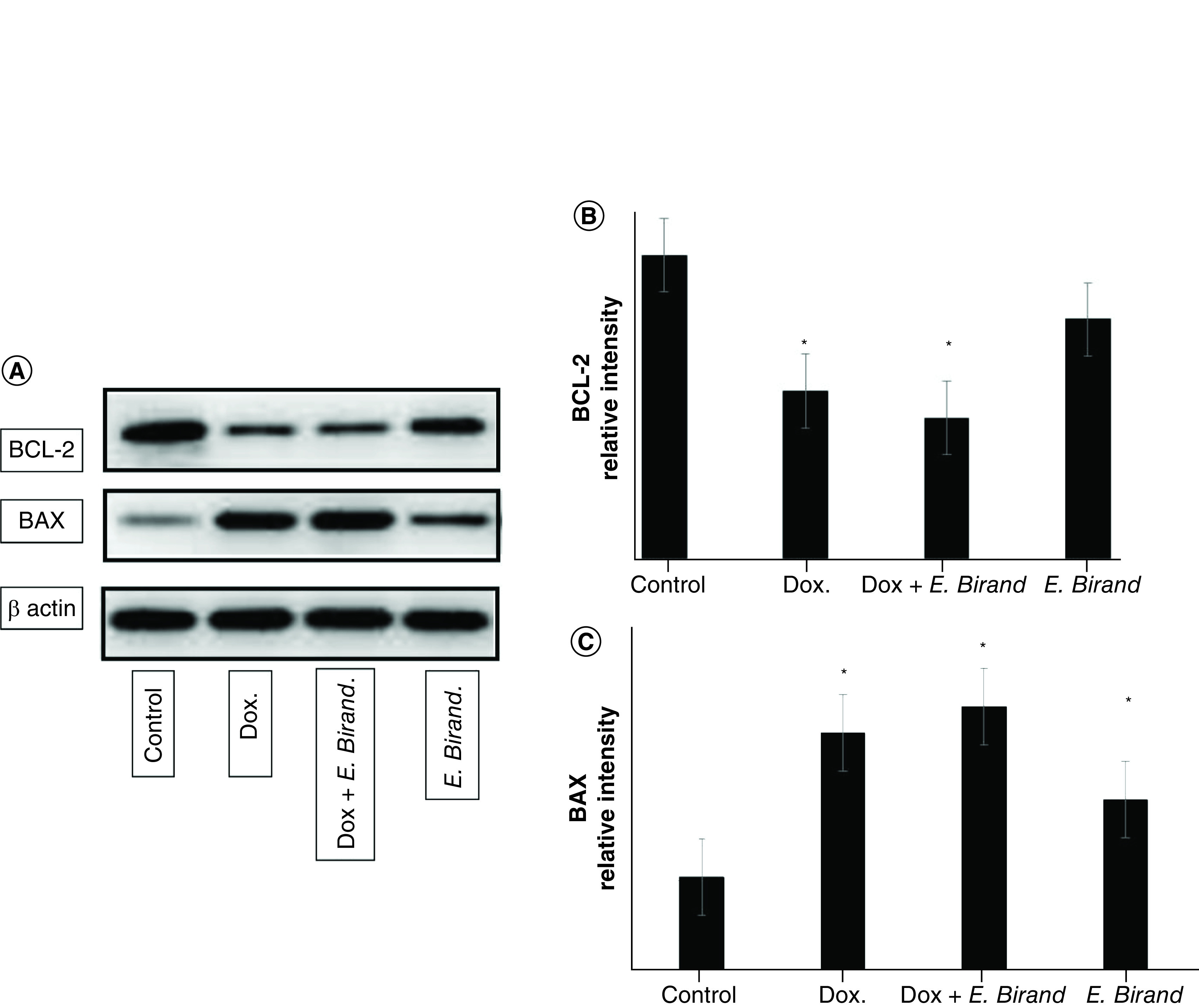
Protein Expressions. **(A)** Levels of expression of BCL-2 and BAX proteins. **(B)** In comparison to negative control, treatment with doxorubicin or combined with *E. birandianum*, significantly reduced the expression of BCL-2. **(C)** Also, *E. birandianum* or in combination with doxorubicin significantly increased the expression levels of BAX protein compared to the control.

The results of the assessment of cellular apoptosis showed that treatment with doxorubicin alone or combined with *E. birandianum*, but not *E. birandianum* alone, significantly reduced the expression of Bcl-2 (antiapoptotic) cells compared with the control (Figure 4A & B). İnterestingly, *E. birandianum* alone or combined with doxorubicine resulted in a significant increase in the expression levels of the proapoptotic BAX protein compared with the control (Figure 4A & C).

## Discussion

Natural products have been the most productive sources of leads for the development of new drugs, especially anticancer agents. Many of today's therapeutic agents owe their existence to natural products, with more than half of all new drug approvals still tracing their structural origins to a natural product [21,22]. Newman *et al.* claimed that 55% of chemotherapeutic drugs approved between 1981 and 2010 were derived from or based on natural products [23]. Thus, natural products will remain one of the most important sources of therapeutic entities for the treatment of various diseases.

In the current study, *in vitro* screening using the cell-based MTT assay was applied to a number of ethanol extracts from the aerial parts of a number of *Erodium* and *Geranium* species against several cancer cell lines. MTT assay is a reliable colorimetric assay commonly used for chemotherapeutic drug screening [20]. The assay measures cellular metabolic activity as an indicator of cell viability, proliferation and cytotoxicity. In principle, metabolically active cells reduce yellow tetrazolium salt (3-(4,5-dimethylthiazol-2-yl)-2,5-diphenyltetrazolium bromide (MTT) to purple formazan crystals; the darker the solution the greater the number of viable cells [24,25]. Interestingly, the results demonstrated that the EtOH extracts of *G. glaberrimum*, *G. asphodeloides*, *E. brandianum*, and *E. leucanthum* were able, with variable potency, to inhibit cellular proliferation of breast cancer cells (MCF-7), muscle Ewing's sarcoma cells (A-673), epithelial cervical carcinoma (DoTc2 4510), hepatocellular carcinoma (HUH-7) and malignant melanoma cells (A-375).

The tested extracts were previously reported to display different biological activities. For example, some of the chemical constituents of *G. glaberrimum* (no. 2), which is endemic to the southwestern region of Turkey [26], were reported to have antiplasmodial and cholinesterase inhibitory activities [27], while the chemical constituents isolated from *E. brandianum* were reported to possess antioxidant and antimicrobial activities [28]. *E. leucanthum* was shown to have antimitotic activity [29]. Interestingly, *G. asphodeloides*, the plant extract with the highest TI, was reported to possess several biological activities including antimicrobial and antioxidant activity [30]. Compared with doxycycline used as the positive control, the extracts showed potent antiproliferative activity against the screened cell lines with *G. asphodeloides* having the most potent therapeutic activity, even higher than that of doxycycline. The extracts showed a similar level of cytotoxicity in nontumor fibroblastic cells (NIH/3T3) to that of the positive control in the concentration range between 20 ng/ml and 20 μg/ml. However, at 200 μg/ml, *E. brandianum* extract was significantly less toxic than the control, whereas *G. glaberrimum* (no. 2) had a similar CC_50_ to the control, but both extracts (*G. asphodeloides* and *E. leucanthum*) displayed high cellular toxicity. While all of the extracts displayed potential antiproliferative activity comparable to the control, the extract of *E. brandianum* exhibited potent antiproliferative activity and low cytotoxicity, resulting in a high TI. Therefore, *E. brandianum* has significant potential for further studies as a potential therapeutic entity not only as chemotherapy but against other diseases that were reportedly treated by other members within the genus *Erodium* L.

Recent phytochemistry analyses of *E. birandianum*, by the authors' group as part of a recent and unpublished PhD thesis led to the identification of a number of phenolics, such as quercetin, as the major component. Other identified components in this plant include gallic acid, caffeic acid, quinic acid, syringic acid, 3,4-dihydroxybenzoic acid, catechin, epicatechin, rutin, luteolin and apigenin (unpublished data by Baki Kekilli) [31]. Therefore, the antiproliferative activity of *E. birandianum* extract shown in the current results is likely associated with one or many of the identified components. The major identified flavonoid, quercetin, was shown to be cytotoxic to MCF-7 cells and, in combination with mycophenolic acid, was in a synergistic manner able to inhibit breast cancer cell growth [32–34]. In a recent study by Yamada *et al.* [35], quercetin was reported to successfully suppress hepatocyte growth factor (HGF) or transforming growth factor-α (TGF-α) in HuH7 cell lines, which was also confirmed by others [36]. Therefore, based on the current finding that quercetin is the major phenolic component in *E. birandianum*, and the recently reported antiproliferative effects of quercetin, we speculate that quercetin is one of the main components responsible for the antiproliferative effect of this particular species.

There are several mechanisms by which these extracts can achieve antiproliferative activity, including autophagy, a cellular process for degrading and eliminating damaged organelles and misfolded proteins that functions in adaptation to cellular development, death and tumor suppression [37]. The autophagic process is controlled by different proteins, including the mammalian target of rapamycin (mTOR), which is associated with cell proliferation and cancer progression [38]. Notably, *G. asphodeloides*, *E. leucanthum* and *G. glaberrimum* (no. 2), but not *E. brandianum*, through as-yet-unknown mechanisms, induced autophagy in MCF7 cells with a similar potency to that of rapamycin (the positive control). Consequently, it can safely be assumed that autophagy initiation is likely to be one of the antiproliferative mechanisms of the active extracts, which is a common mechanism seen in the PI3K/Akt/mTOR axis-inhibiting anticancer drugs [39].

Another interesting mechanism that was tested is the induction of the apoptotic pathway, which is thought to be an effective method for the treatment of cancer, as the cell numbers are dependent upon the extent of cell proliferation and death [35]. However, there are several mechanisms that cancer cells use to evade apoptosis, including the overexpression of the antiapoptotic Bcl2 family of proteins, a protein family that regulates the mitochondrial cell-intrinsic apoptotic pathway [36,37]. The Bcl2 protein binds to proapoptotic members, such as BAX, a protein that induces cell death in tumor cells, to prevent pore formation and cytochrome c release [38]. Cell treatment with doxorubicin alone or combined with *E. birandianum* resulted in markedly lower levels of Bcl2 protein expression compared with the control. Treatment with *E. brandianum* alone also resulted in a statistically insignificant reduction in the protein expression level of Bcl2 compared with the control. In contrast, cell treatment with doxorubicin alone, *E. brandianum* alone or combined increased the expression level of BAX protein compared with the control. These data show that cellular treatment with *E. brandianum* can alter the ratio of Bcl2 and BAX proteins, which is an important step in deciphering the potential antiproliferative effects of *E. brandianum*. Thus, it is safe to suggest that *E. brandianum* is an inducer of cellular apoptosis through changes in the protein expression ratio of Bcl2 and BAX.

The results in the present study describe the potential antiproliferative activities of 4 out of 21 tested plant extracts from several *Geranium* and *Erodium* species. The data demonstrated remarkable antiproliferative activity of the four plant extracts against five different cancer cell lines with overall TI values comparable to those of the positive control, doxycycline. Although the exact mechanisms of action of these extracts are not fully determined, all of the extracts, except *E. brandianum*, seemed to induce cellular autophagy in tumor cells with similar levels to that of rapamycin. Also, *E. brandianum* appears to induce cellular apoptosis mediated through Bcl2 and BAX protein expressions.

## Conclusion

The current study is a proof of concept that medicinal plants can provide a substantial number of drug lead candidates with various biological activities. This is the first study to report the potential anticancer effects of ethanol extracts of the *Geraniaceae* species. Only 4 of the 21 screened species, *G. asphodeloides*, *G. glaberrimum* (no. 2), *E. leucanthum* and *E. birandianum* displayed antiproliferative activities in a dose-response manner against different cancer cell lines. While the majority of the screened species induced cellular autophagy in tumor cells, only *E. brandianum* caused apoptosis. Further studies are required to identify the bioactive components of the extracts that showed antiproliferative effects and to determine their mechanisms of action.

**Summary points**

- There is a need for effective and less toxic anticancer drugs.
- Medicinal plants represent an invaluable source of therapeutic agents, including anticancer drugs.
- Geraniaceae is a plant genus that comprises more than 400 species.
- A number of plant species have therapeutic uses and have been used in folk medicine in different regions.
- Five cancerous cell lines were used to test the antiproliferative potential of the ethanol-extracted species.
- Only four of the extracts showed potential antiproliferative activity in a dose-response manner.
- The noncancerous cell line NIH/3T3 was used to determine the cytotoxic effects.
- *E. birandianum* was shown to have a higher therapeutic index than doxycycline.
- In combination with doxorubicin, *E. birandianum* significantly reduced the expression of Bcl-2 and increased the expression level of BAX, compared with control.
- Natural products are a good source of drug leads.
- *In vitro* screening of *Erodium* and *Geranium* species using MTT assay against five different cell lines showed potential antiproliferative activities.
- Four of the extracts showed low toxicity compared with the control.
- The polyphenol quercetin is a major component of *E. birandianum*, which may be responsible for the observed antiproliferative potential.
- The potential antiproliferative mechanism of action of *G. asphodeloides*, *E. leucanthum* and *G. glaberrimum* (no. 2) might be facilitated by autophagy.
- The potential mechanism of action of *E. birandianum* is likely to be mediated through its apoptotic potential, as shown by its ability to decrease Bcl-2 protein expression and increase the BAX protein expression compared with the control.
